# Effects of CYP46A1 Inhibition on Long-Term-Depression in Hippocampal Slices *ex vivo* and 24S-Hydroxycholesterol Levels in Mice *in vivo*

**DOI:** 10.3389/fnmol.2020.568641

**Published:** 2020-10-29

**Authors:** Michael Popiolek, Yukitoshi Izumi, Allen T. Hopper, Jing Dai, Silke Miller, Hong-Jin Shu, Charles F. Zorumski, Steven Mennerick

**Affiliations:** ^1^Sage Therapeutics, Cambridge, MA, United States; ^2^Department of Psychiatry, Taylor Family Institute for Innovative Psychiatric Research, Washington University, University School of Medicine in St. Louis, St. Louis, MO, United States

**Keywords:** CYP46A1, 24S-hydroxycholesterol, cholesterol, LTD, NMDAR

## Abstract

The manipulation of cholesterol and its metabolites has been hypothesized to be therapeutically beneficial for mood disorders, neurodegenerative disorders, and epilepsies. A major regulator of cholesterol clearance and turnover in the central nervous system is CYP46A1, a brain enriched enzyme responsible for metabolism of cholesterol into 24S-hydroxycholesterol. Inhibition of this enzyme may negatively modulate NMDARs as 24S-hydroxycholesterol was shown to enhance NMDAR function. In addition, alterations of local cholesterol or other changes mediated by CYP46A1 activity could have important influences on central nervous system function. Here we demonstrate that humans and mice display brain region specific and similar CYP46A1 and 24S-hydroxycholesterol distribution. Treatment with distinct classes of CYP46A1 inhibitors led to central 24S-hydroxycholesterol reduction *in vivo* and ablation of long term depression in hippocampal slices. Our results suggest that rodents show similarity to humans for studying the impact of CYP46A1 inhibitors and that rapid, local modulation of oxysterols can be achieved through CYP46A1 inhibition.

## Introduction

The oxysterol 24S-hydroxycholesterol (24S-HC) is an enzymatically generated oxidation product of cholesterol. It is selectively synthesized in central nervous system (CNS) neurons by the cytochrome P450 enzyme cholesterol 24-hydroxylase (*CYP46A1*). Although 24S-HC flux to the periphery is important for brain cholesterol turnover (Russell et al., 2009), signaling roles for 24S-HC and therapeutic interventions targeting 24S-HC in the CNS have also been suggested (Alves et al., 2016; Sun et al., 2016b; Bialer et al., 2018; Petrov and Pikuleva, 2019). Better understanding of the roles of 24S-HC requires knowledge of regional variation in CYP46A1 and 24S-HC levels, as well as how manipulations of 24S-HC levels impact brain function.

The brain synthesizes its own cholesterol, as lipoprotein-bound cholesterol does not pass the blood-brain barrier. The neuronal cholesterol pool is labile and turns over regularly, primarily by conversion to 24S-HC, which passes the blood-brain barrier for elimination in the liver (Russell et al., 2009). Interestingly, constitutive loss of CYP46A1 does not result in changes to cholesterol concentrations or in other oxysterols (Meljon et al., 2014). This implies complex regulatory and feedback mechanisms to maintain cholesterol homeostasis. However, acute changes to CYP46A1 function alter cholesterol and 24S-HC levels (Sodero et al., 2012; Djelti et al., 2015; Bialer et al., 2018; Kacher et al., 2019), which could have an impact on brain physiology.

It has been shown that many cholesterol metabolites, including oxysterols, have CNS signaling roles (Paul et al., 2013; Sun et al., 2017). 24S-HC appears to be no exception. It alters transcription by binding liver X receptors (Gabbi et al., 2014), and modulates ion channel function such as the N-methyl-D-aspartate receptor (NMDAR) (Paul et al., 2013). 24S-HC elevation may have a therapeutic effect in neurodegenerative conditions where decreased levels of CYP46A1 enzyme have been reported (Alves et al., 2016), but inhibition of CYP46A1 may be beneficial in diseases accompanied by increased levels of 24S-HC, such as hypoxia (Lu et al., 2020), conditions that promote excessive excitation, which are associated with increased CYP46A1 activity (Sodero et al., 2012). Regional variability in 24S-HC levels may yield insight into the impact of 24S-HC on brain function and behavior, but little work has been done to evaluate this notion (Yutuc et al., 2019, 2020).

Here we evaluated regional expression and distribution of CYP46A1 and 24S-HC in mouse and compared it to published data from human brain. We followed up by characterizing known structurally distinct CYP46A1 inhibitors. We find selective actions of these compounds *ex vivo* and *in vivo* which we attribute to CYP46A1 inhibition as they have no demonstrable binding at a number of other potential protein targets. Systemic administration of these compounds achieved good brain exposure and reduced 24S-HC. However, administration of inhibitors for up to 6 h to hippocampal tissue slices did not appreciably alter 24S-HC levels but altered long-term synaptic plasticity, suggesting that local actions of 24S-HC may be important for NMDAR mediated function. Our results help establish a more comprehensive picture of potential therapeutic applications of 24S-HC synthesis pathways.

## Materials and Methods

### Compound Supply

Structurally distinct CYP46A1 inhibitors, compound 1 ((3-oxa-8-azabicyclo[3.2.1]octan-8-yl)(8-(oxazol-5-yl)-6-(trifluoromethyl)imidazo[1,2-a]pyridin-3-yl)methanone (Kamal et al., 2015) and compound 2 ((4-(4-fluorobenzyl)-4-hydroxypiperidin-1-yl)(2-(pyrimidin-4-yl)pyridin-3-yl)methanone, were identified (Figure 3A) and synthesized. Voriconazole (Bioscience, Ellisville MO), ketamine hydrochloride injectable (Gutian pharmaceutical Co. Ltd., Fujian), hydroxypropylmethyl-cellulose (MC; Sigma-Aldrich, St. Louis, MO, United States; CAS#9004-65-3) and sterile saline (Shan Dong Hualu Pharmaceutical, Shan Dong, China) were purchased.

### Data Mining From the GTEx Portal

Human sample collection and analysis for mRNA quantification by the GTEx portal has previously been described (Lonsdale et al., 2013). The mean number of subjects examined for CYP46A1 expression within samples presented was 317 with the maximum examined in muscle from 803 subjects and minimum in kidney from 4 subjects. Within the brain, the lowest number of samples was collected from substantia nigra where 139 subjects were examined.

### Western Blot

Proteins were isolated from mouse brain tissue with RIPA lysis buffer containing 150 mM sodium chloride, 1% Triton X-100, 0.5% sodium deoxycholate, 2.5 mM EDTA, 0.1% SDS, and 50 mM Tris-HCl, pH 8, with protease inhibitor cocktail (MilliporeSigma, St. Louis, MO). Homogenates were centrifuged at 15,000 rpm at 4°C for 20 min. The clear supernatant was stored at −70°C. Protein concentrations within each homogenate were determined with the Pierce Coomassie protein assay reagent (Micro BCA, Thermo Fisher Scientific, Waltham, MA). A 25 μg protein sample was run on 4–15% polyacrylamide gel (Bio-Rad Laboratories, Hercules, CA) at 100 V for 1 h. Proteins and molecular weight markers (Lamda Biotech Corp., St. Louis, MO) were transferred by electrophoresis onto an Immunobilon-P membrane (MilliporeSigma, St. Louis, MO) at 4°C and processed for immunodetection. Incubation of the membrane with blocking solution (3% non-fat milk in PBS for 1 h at RT) was followed with incubation in primary antibody (Rabbit anti CYP46A1, #SAB-1410134; MilliporeSigma) at 1 μg/mL dilution in blocking solution at 4°C overnight and washed three times. The membrane was next incubated with horseradish peroxidase-conjugated goat anti-rabbit IgG secondary antibody (Santa Cruz Biotechnology, Dallas, TX) 1:2,000 dilution for 2 h. A Lumigen ECL Ultra kit was used for detection. Bands were digitized on a Kodak ImageStation 440CF. The intensity of bands was quantitated with ImageJ software (NIH). After image capture, blots were stripped using Restore Western blot Stripping Buffer (Thermo Fisher Scientific) and reprobed with tubulin antibody (MilliporeSigma) as an internal control for sample loading.

### CYP46A1 Enzyme Assay With Testosterone as Substrate

In 96-well plate format, 88.5 μL of human CYP46A1R bactosomes (50 pM/mL final concentration; Cypex, Catalog Number CYP068) in potassium phosphate buffer with MgCl_2_ and 1 μL of varying half-log concentrations of inhibitor or a standard curve of 16β-hydroxytestosterone were added to designated wells and incubated at 37°C for 2 min. The substrate testosterone (0.5 μL of a 3,000 μM solution) and 10 μL of the NADPH generating system were added to all wells and incubated for 10 min at 37°C. The reaction was stopped by adding 100 μL of methanol containing 1 μM of the internal standards bucetin and diclofenac. The plates were sealed and placed in a −20°C freezer for 10 min, centrifuged at 5,700 rpm for 20 min at 4°C and the supernatants transferred for LC-MS/MS analysis.

The NADPH generating system was prepared by combining 1,500 μL of 200 mM glucose-6-phospate, 3,000 μL of 20 mM NADH, 60 μL of 1,000 U/mL glucose-6-phosphate dehydrogenase and 1,440 μL of 100 mM potassium phosphate at pH 7.4.

### 16β-Hydroxytestosterone LC-MS Analysis

Samples were injected onto a Waters Acquity UPLC with an Acquity UPLC HSS T3 1.8 μm, 2.1 × 50 mm column and triple quadrupole Xevo TQ MS detector using a gradient elution using mobile phase A of water with 0.1% formic acid and mobile phase B of acetonitrile with 0.1% formic acid. The gradient was maintained at 2% B for 0.05 min, then ramped linearly to 95% B in 0.95 min, then immediately returned linearly to 2% B in 0.50 min with a total runtime of 1.8 min. The reaction product 16β-hydroxytestosterone was monitored (parent m/z 305.1844 and daughter m/z 96.9690) and quantified. Dose response curves were generated by graphing 16β-hydroxytestosterone levels vs. inhibitor concentrations.

### CYP46A1 Cholesterol Assay

In 384-well plate format, 0.5 μL of human CYP46A1 microsomes (25mg/mL, Premas Biotech, Gurugram, India) were incubated for 5 min with 2-hydroxypropyl-β-cyclodextrin (10 mg/mL) and 100 mM potassium phosphate buffer (pH 7.4) containing 5 mM MgCl_2_. 1 μL of varying half-log concentrations of inhibitor were added to designated wells and incubated for 5 min. Cholesterol (25 μM) was then added and the reaction was initiated using 1 mM NADPH. The reaction was conducted at 37°C for 30 min in a total incubation volume of 50 μL. After the incubation 30 μL of incubation mixture was added to a pre-made quench plate containing 30 μL 1 μM d7-24 hydroxy cholesterol in acetonitrile/methanol (50/50) to terminate the reaction. Subsequently, the plates were sealed and placed in a −20°C freezer for 10 min, centrifuged at 5,700 rpm for 20 min at 4°C and the supernatants transferred for LC-MS/MS analysis.

### 24-Hydroxycholesterol LC-MS Analysis

Samples were injected onto a SCIEX 6500 QTRAP Mass Spectrometer with an Eclipse XDB-C18 (3 × 100 mm, 3.5 μm) column using a gradient elution using mobile phase A of water with 0.1% formic acid and mobile phase B of methanol:acetonitrile (50:50, v:v) at a flow rate of 600 μL/min at 50°C. The gradient was maintained at 80% B for 0.01 min, then ramped linearly to 100% B in 3 min, then returned linearly after 3.3 min to 80% B in 4.30 min with a total runtime of 11 min. The reaction product 24-hydroxycholesterol was monitored (parent m/z 385.0 and daughter m/z 367.0) and quantified in comparison to d7-24-hydroxycholesterol standard. Dose response curves were generated by graphing 24-hydroxycholesterol levels vs. inhibitor concentrations.

#### Analytical Method for 24S-HC Quantification in Mouse Plasma, Brain, and Brain Slices

Male CD1 mice (Vital River, Beijing, China) were housed in plastic cages with metal covers (GAU-2, Suzhou Fengshi Laboratory animal equipment Co., LTD, Suzhou, China) in groups of 5 under controlled conditions (temperature of 20−26°C and 12:12 light-dark cycle, lights on at 5:00 am) with a standard rodent diet and water available *ad libitum*. The mice were acclimated for 5 days under these conditions before testing. The animals were randomly assigned to experimental groups; each mouse was used only once. At the time of the experiment mice were 5–6 weeks old. The experiment was approved by the Institutional Animal Care and Use Committee of Shanghai ChemPartner Co., Ltd.

Under the assay conditions, both 24S and 24R-hydroxycholesterol were detected in a single peak and quantified as total 24-hydroxycholesterol. Total 24-HC was measured using ester hydrolysis, liquid-liquid extraction, and measurement by LC-MS/MS. Brain or plasma samples were first diluted with PBS at a ratio of 3 mL of PBS to 1 g of tissue. Tissue homogenate was prepared using a bead mill type homogenizer (Spex-certiprep Genogrinder).

The 50 μL sample with 5 μL of 24-hydroxycholesterol-d6 internal standard was added to 100 μL of 0.35 N KOH in methanol and heated to 80°C for 2 h. Hydrolyzed plasma or tissue homogenate (155 μL) was mixed with 200 μL of DI water and extracted with 1 mL of methyl-t-butyl ether. The organic layer was separated and taken to dryness under nitrogen at 50°C. The dry residue was reconstituted in 100 μL of 80% methanol in DI water. Reconstituted sample (5 μL) was injected on an ACE 3C18 0.5 mm × 1.0 mm HPLC column. Gradient elution was performed using an Eksigent LC200 HPLC system running a simple water:acetonitrile gradient from 70% acetonitrile to 95% acetonitrile over 4.75 min at a flow rate of 100 μL/min.

Calibrators and assay quality controls were made by spiking 24S-hydroxycholesterol-d7 into control mouse plasma or brain homogenate and preparing them as samples. Detection of 24S-HC was accomplished using a Sciex Qtrap 5500 mass spectrometer running selected reaction monitoring of the analyte and internal standard. Ions were formed using a TurboV electrospray ion source operated in the positive ion mode. Sample concentrations were determined using the peak area ratio of analyte to internal standard and the least squares linear regression equation from the standard curve.

Assay acceptance criteria for each LC-MS/MS run were ±20% accuracy compared to the nominal spiked concentration and ±20% CV. Endogenous quality controls made from control plasma or brain tissue were used to track longitudinal assay performance. Endogenous quality controls must reproduce within 3 SDs of the latest 20 measurements.

### Hippocampal Slice Physiology

Protocols for animal use in *ex vivo* studies were approved by the Washington University Institutional Animal Care and Use Committee in accordance with international guidelines. Hippocampal slices were freshly prepared from the dorsal hippocampal region of postnatal day (P) 30–34 Harlan Sprague-Dawley male albino rats or C57Bl/6 mice (Tokuda et al., 2010). CYP46A1 mice were on the C57B/6 background as described previously (Sun et al., 2016a). Rodents were anesthetized with isoflurane, decapitated, and hippocampi were dissected. Isolated hippocampi were placed in ice-cold artificial cerebrospinal fluid (ACSF) containing (in mM): 124 NaCl, 5 KCl, 2 MgSO_4_, 2 CaCl_2_, 1.25 NaH_2_PO_4_, 22 NaHCO_3_, 10 glucose, bubbled with 95% O_2_-5% CO_2_ at 4−6°C, and cut into 450 μm slices using a rotary tissue slicer. Following dissection, slices were allowed to recover in an incubation chamber containing gassed ACSF for 1 h at 30°C before experiments.

At the time of study, slices were transferred individually to a submersion-recording chamber that was maintained at 30°C with continuous ACSF perfusion at 2 mL/min. Extracellular recordings were obtained from *stratum radiatum* of the CA1 region for analysis of excitatory postsynaptic potentials (EPSPs) using glass electrodes filled with 2 M NaCl (5–10 MΩ resistance).

EPSPs were evoked using 0.1 ms constant current pulses through a bipolar stimulating electrode in the Schaffer collateral (SC) pathway. Responses were monitored throughout experiments using single stimuli every 60 s at half maximal intensity based on a control input-output (IO) analysis. After establishing a stable baseline for at least 10 min, long-term depression (LTD) was induced using 1 Hz low frequency stimulation (LFS) for 15 min (900 pulses), a stimulation protocol that reliably induces NMDAR-dependent LTD (Izumi and Zorumski, 2012). IO curves were repeated 60 min following LFS.

### Statistical Analysis

All data presented as mean ± S.E.M. Graphpad Prism 8.0 was used for all data analyses. A one-way ANOVA followed by Dunnett’s test was used for analysis of CYP46A1 protein expression (Figure 2B) and 24S-HC levels (Figure 2C) in the mouse brain regions. Indicated where multiple comparison significance (*p* < 0.05 or better) between experimental variables. A two-way ANOVA with multiple comparison was used for assessment of compound 1 on 24S-HC at indicated timepoints (Figure 4). A two-way ANOVA (dose and time as between factors) evaluated the time course of compound 1 compared to vehicle across the selected time points (Figure 6). After a significant interaction between dose and time was identified, a Sidak’s multiple comparison test was used to evaluate vehicle vs. compound 1 at each timepoint. Measurements from two female mice did not obviously differ from those of males, so we did not pursue sex differences further (Supplementary Figure 1).

Electrophysiogical data were collected and analyzed using PClamp software (Axon Instruments, Union City CA). Data in the text are expressed as mean ± SEM. A two-tailed Student’s *t*-test was used for comparisons between groups with correction for multiple comparisons when appropriate. Statistical comparisons were based on data from IO curves at baseline and 60 min following LFS with *p* < 0.05 considered significant. The graphs in all figures display results from continuous monitoring of synaptic responses at very low frequency during the course of experiments, while results presented in the text and statistical comparisons are derived from analysis of IO curves as noted above. Statistical analyses were done using commercial software (SigmaStat, Systat Software, Inc., Richmond City, CA).

## Results

### CYP46A1 Expression Within Human and Rodent Brains

**FIGURE 1:**
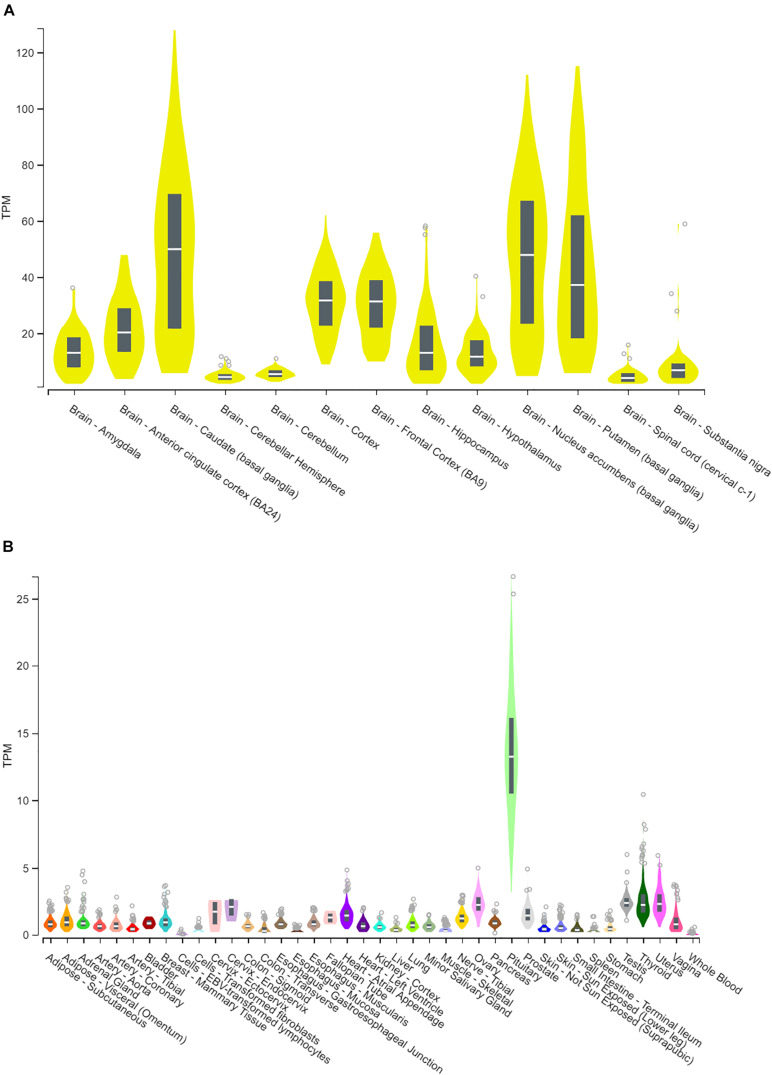
Human CYP46A1 expression, from RNA-seq analysis. CYP46A1 mRNA is predominantly expressed within the brain **(A)** but not in the periphery **(B)**. Brain expression is highest in the striatum > cortex > hippocampus > cerebellum. Box plots are shown as median with 25th and 75th percentiles. TPM, transcripts per million. Data mined from GTEx portal (Lonsdale et al., 2013). The Genotype-Tissue Expression (GTEx) Project was supported by the Common Fund of the Office of the Director of the National Institutes of Health, and by NCI, NHGRI, NHLBI, NIDA, NIMH, and NINDS. The data used for the analyses described in this figure were obtained from the GTEx Portal on July 16th of 2019.

Human CYP46A1 expression, based on RNA-seq quantification, is preferentially expressed within the central nervous system, where transcripts are in excess of 100 per million while in most of the peripheral tissues they are less then 5 transcripts per million (Figure 1). The only notable exception is the pituitary gland where CYP46A1 message can be as high as 25 transcripts per million (Figure 1B). Within the brain, CYP46A1 exhibits differential tissue expression as its distribution is highest within the striatum (caudate, putamen and nucleus accumbens), cortex (cortex, frontal cortex and anterior cingulate cortex), amygdala, hippocampus and cerebellum (Figure 1A). Human RNA-seq data were mined from the GTEx Portal.

**FIGURE 2:**
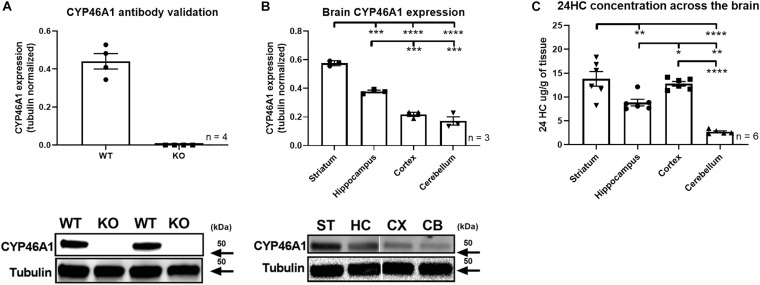
Mouse brain CYP46A1 and 24S-HC expression. CYP46A1 antibody was validated on whole brain lysate from CYP46A1 KO mice **(A)**. Topographically distinct CYP46A1 rodent brain expression observed with highest expression in the striatum > hippocampus > cortex/cerebellum. Representative lanes from western blots are shown. **(B)** Brain region 24S-HC differential observed with highest 24S-HC detected in striatum/cortex > hippocampus > cerebellum **(C)**. *p < 0.05; **p < 0.01; ***p < 0.001; ****p < 0.0001.

In mouse, we used an antibody to quantify CYP46A1 protein expression. Similar to human, mouse CYP46A1 distribution displays brain region specific expression. CYP46A1 antibody was first confirmed to be immunoreactive against whole brain lysates from wildtype, but not CYP46A1 knockout mice, on western blot (Figure 2A), reinforcing its selectivity for CYP46A1 detection. This antibody was subsequently used to quantify CYP46A1 protein expression across several mouse brain regions, including the striatum, hippocampus, cortex, and cerebellum (Figure 2B). We found this CYP46A1 antibody not to be suitable for immunohistochemistry staining, as judged by staining in postnatal day 70–90 knockout tissue (Sun et al., 2016a) under several different fixation and permeabilization protocols (Supplementary Figure 2). Other antibodies have been used previously for immunostaining (Ramirez et al., 2008), but in our hands the 1A7 antibody used in this work (MilliporeSigma) yielded multiple bands in western blots, so we did not pursue it.

Since the presumed function of the CYP46A1 enzyme is to catalyze synthesis of 24S-HC (Mast et al., 2004) we also quantified this product across several mouse brain regions. 24S-HC was highest in the striatum and lowest in the cerebellum (Figure 2C) which reflected CYP46A1 expression levels. The cortical 24S-HC level was similar to that of the striatum and higher than expected based on CYP46A1 protein expression, suggesting different rates of synthesis or retention of this oxysterol. Mouse gender did not have an obvious impact on the level of 24S-HC (Supplementary Figure 1).

### *In vitro* and *in vivo* Pharmacology of CYP46A1 Inhibitors

**FIGURE 3:**
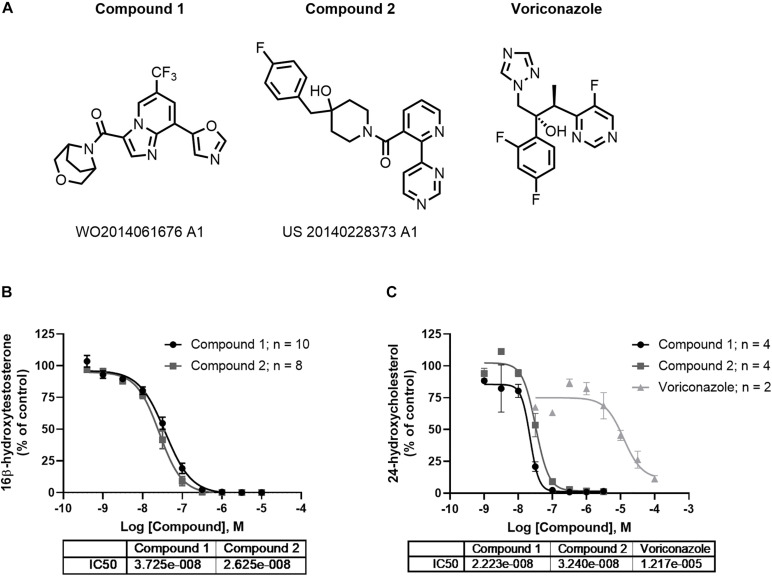
*In vitro* profile of CYP46A1 inhibitors. Compounds 1 and 2 are examples of chemically distinct **(A)** and potent inhibitor classes that completely inhibit CYP46A1 when used with testosterone **(B)** or cholesterol as substrates **(C)**. The numbers beneath the compounds indicate patent numbers.

Compounds 1 and 2 represent examples of two distinct structural classes of CYP46A1 inhibitors identified recently (Figure 3A). These examples have been of interest as tool compounds in commercial development (indicated by patent numbers in Figure 3). Both representatives completely inhibit the oxidation of testosterone to 16-beta-hydroxytestosterone by human CYP46A1 enzyme *in vitro* with IC_50s_ of 26 and 37 nM, respectively (Figure 3B). *In vitro* potency of voriconazole using testosterone as a substrate was found to be 35.9 μM (Figure 3B). Initially, testosterone was utilized as substrate in this reaction owing to its improved solubility over cholesterol, enabling better run to run reproducibility (Mast et al., 2003). We also developed an *in vitro* assay using cholesterol as a substrate. Both compounds 1 and 2 completely inhibit the conversion of cholesterol to 24-hydroxycholesterol by human CYP46A1 enzyme *in vitro* with IC_50s_ of 22 nM and 32 nM (Figure 3C), comparable to the potency using testosterone as substrate (Figure 3B). The IC_50_ of voriconazole in the cholesterol assay was 12.2 μM (Figure 3C). Both compounds 1 and 2 were found to be free of off-target pharmacology as demonstrated by lack of meaningful effect against a series of 69 receptor and enzyme targets with in a CEREP panel (Supplementary Table 1), 7 CYP enzymes (Supplementary Table 2) and 97 kinases (Supplementary Table 3).

**FIGURE 4:**
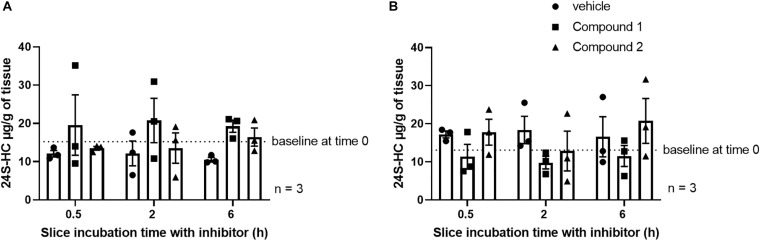
No impact of CYP46A1 inhibitor treatment on striatal **(A)** or hippocampal **(B)** slice 24S-HC levels. The number of independent tissue slices from three different animals is given. Tissue harvest followed by rapid freezing occurred at the indicated times.

In an attempt to further elucidate the properties of these CYP46A1 inhibitors, we were curious to see if we could decrease 24S-HC levels in brain slices after acute administration of CYP46A1 inhibitors. Application of either of the two CYP46A1 inhibitors to mouse striatal and hippocampal slices for 0.5, 2, or 6 h did not influence bulk 24S-HC levels within those slices relative to vehicle treatment (Figure 4). Possibilities for the lack of an effect could be due to low aqueous solubility of 24S-HC preventing washout of existing hydroxycholesterol, insufficient time of CYP46A1 inhibition or a species difference, which would preclude these inhibitors from working on the mouse CYP46A1 enzyme.

**FIGURE 5:**
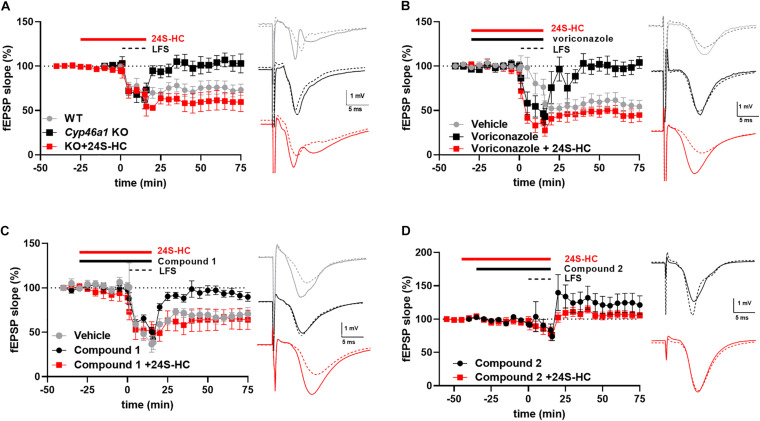
The impact of various inhibitors of CYP46A1 on long-term depression in hippocampal slices. **(A)** Constitutive deletion of *cyp46a1* in mice results in loss of LTD. Exogenous application of 24S-HC (10 μM) restores LTD. For WT, LTD as a percentage of baseline efficacy was 71.1 ± 7.3%, *N* = 8; KO: 94.7 ± 8.3%, *N* = 9, *p* = 0.049. 24S-HC: 54.6 ± 8.4%, *N* = 5, *p* = 0.007 vs. KO without 24S-HC. Sample traces for this and subsequent panels show baseline (solid) and post-LFS (dashed) examples, color-coded as in the time plot. **(B)** Voriconazole (3 μM) reduces LTD vs. vehicle controls (*p* = 0.0027), and 10 μM 24S-HC restores LTD in rat slices. LTD was 104.9 ± 5.3%, *N* = 5 in the presence 3 μM voriconazole alone and 53.8 ± 12.4% (*N* = 5) with pretreatment with 24S-HC (10 μM), *p* = 0.006. **(C,D)** Compound 1 (10 μM; 97.9 ± 7.7%, *N* = 5, *p* = 0.0039 vs. vehicle controls) and compound 2 (1 μM; 129.0 ± 13.4%, *N* = 7, *p* = 0.0014 vs. vehicle) reduce LTD; 10 μM 24S-HC restores LTD only for compound 1 (10 μM; 66.8 ± 10.4%, *N* = 6, *p* = 0.046; for 24S-HC + compound 2 105.5 ± 6.7%, *N* = 7, *p* = 0.144). Concentrations of compounds 1 and 2 were chosen based on threshold effects on LTD; LFS with Compound 1 at 1 μM yielded EPSPs 48.9 ± 9% of baseline (*n* = 5). Compound 2 at 0.1 μM yielded EPSPs 48.2 ± 5.8% of baseline (*n* = 5). Vehicle control data in C were used for comparisons in D, as the experiments were performed contemporaneously.

To help understand these possibilities, we turned to NMDAR-dependent forms of synaptic plasticity. In postnatal day 30 CYP46A1^–/–^ mice, we previously found no deficit in hippocampal CA1 LTP (Sun et al., 2016a). Rather, a significant LTD deficit in hippocampal slices from CYP46A1 knockout mice was observed (*p* = 0.049) (Figure 5A), and we previously showed that synaptic depression triggered by oxygen-glucose deprivation is dependent on CYP46A1 (Sun et al., 2016a). Voriconazole, a previously characterized pharmacological inhibitor of CYP46A1 (Shafaati et al., 2010; Mast et al., 2013), also depressed LTD (*p* = 0.0027) in a 24S-HC sensitive manner (*p* = 0.006) (Figure 5B). The emerging picture was somewhat more complicated with the newer CYP46A1 inhibitors examined here. Both compounds inhibited LTD (*p* = 0.0039 and 0.0014, respectively). However, 24S-HC overcame the LTD deficit induced by compound 1 (*p* = 0.046) (Figure 5C) but failed to reverse the LTD deficit induced by compound 2 (*p* = 0.144) (Figure 5D). Pharmacologically isolated NMDA receptor-mediated EPSPs were unaffected by compounds 1 and 2, and by voriconazole at concentrations that inhibited LTD (Supplementary Figure 3). Potential reasons for the differing behavior of the compounds are treated in the Discussion.

**FIGURE 6:**
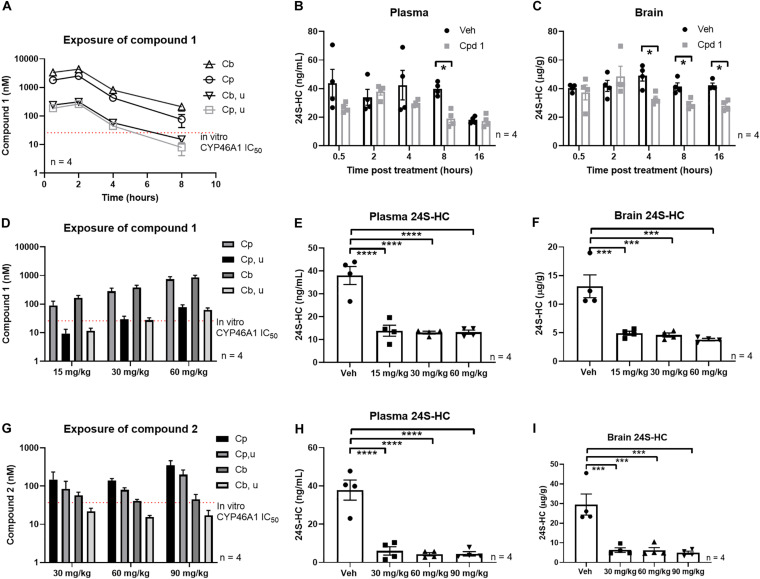
Systemic treatment with CYP46A1 inhibitor decreased central and peripheral 24S-HC. Mouse PK after single systemic administration of 30 mg/kg of compound 1 **(A)**. Significant plasma 24S-HC reduction observed 8 h after compound 1 administration **(B)**. Brain 24S-HC reduction observed between 4 and 16 h after CYP46A1 inhibitor treatment **(C)**. Mouse PK after 3 days of BID administration of compound 1 at 15, 30, and 60 mg/kg doses **(D)**. Significant plasma **(E)** and brain **(F)** 24S-HC reduction observed with every dose. Mouse PK after 3 days of BID administration of compound 2 at 30, 60, and 90 mg/kg doses **(G)**. Significant plasma **(H)** and brain **(I)** 24S-HC reduction observed with every dose. All observations with compound 1 and 2 in the subchronic studies were made 4 h after last compound administration. Cpd 1, compound 1; Cb, total brain concentration, Cp, total plasma concentration, Cb, u, unbound brain concentration, Cp, u, unbound plasma concentration. *p < 0.05; ***p < 0.001; ****p < 0.0001.

Single oral administration of compound 1 to mice led to peripheral and central exposure in excess of the *in vitro* CYP46A1 IC_50_ for at least 4 h (Figure 6A). Since we observed sufficient exposure of compound 1 to suggest engagement of CYP46A1, we quantified the impact of acute treatment on plasma and brain 24S-HC within 16 h of treatment. While vehicle treated plasma 24S-HC underwent some changes during the time of the experiment, perhaps suggesting a level of experimental variability, a statistically significant plasma 24S-HC decrease was observed 8 h post compound 1 treatment (Figure 6B). A statistically significant decrease in brain 24S-HC was also observed after CYP46A1 inhibitor administration at 4, 8, and 16 h (Figure 6C) demonstrating that inhibition of CYP46A1 *in vivo* results in decreased 24S-HC levels in both brain and plasma within a relatively short timeframe.

Next, we utilized a subchronic paradigm, consisting of 3 days of BID dosing, to test if further 24S-HC reductions could be observed with a longer duration of CYP46A1 inhibition. We also tested if compound 2 would likewise lead to similar 24S-HC reductions. Longer exposure of the two compounds, at the same dose as in the acute study (Figures 6D,G), resulted in significantly larger plasma (Figures 6E,H) and brain (Figures 6F,I) 24S-HC reduction than with the acute treatment.

Plasma 24S-HC levels are about the same level, reaching about 40 ng/mL, in mice from the three vehicle treated experiments (Figures 6B,E,H). However, vehicle treated brain 24S-HC levels display considerable variability at 40, 13, and 29 μg/g in the three experiments (Figures 6C,F,I), respectively. This variability may be attributed to the nature of the matrix, and its propensity for endogenous interferences in tissue bioanalysis. Brain is a fatty tissue enriched in endogenous lipophilic components, presenting challenges in extracting out the similarly lipophilic 24S-HC analyte from other cholesterol-like molecules. Matched vehicle controls were used in every experiment to accurately assess magnitude of treatment mediated 24S-HC changes to control for inter-assay variability. Further work may be needed to lessen the variability and improve the extraction analytics.

## Discussion

Cholesterol oxidation by CYP46A1 is the putative mechanism responsible for cholesterol clearance from the brain (Russell et al., 2009). Hence, it is not surprising that CYP46A1 expression is predominantly localized to the brain (Figure 1). Although mRNA transcription generally leads to protein synthesis, this is not always the case, and assumption of a correlation may not be warranted. Nonetheless, we found that within human and rodent brain CYP46A1 mRNA and protein expression are similar in critical brain regions across species, while displaying regional variability with most abundant expression in striatum and least abundant in cerebellum (Figures 1A, 2B). Furthermore, 24S-HC levels, within those two brain regions, appear to mirror CYP46A1 expression (Figure 2C) suggesting a similar enzymatic specific activity. These observations, supported by previously published results in CYP46A1 knockout mice (Ramirez et al., 2008; Meljon et al., 2014), raise the possibility of differential rates of cholesterol turnover or different mechanisms, beyond CYP46A1, responsible for maintaining cholesterol homeostasis among different brain regions. None of the less, having observed an effect on 24S-HC raises possibility of also impacting cholesterol levels. Although we did not quantify cholesterol at baseline or in different brain regions, we do not expect to have had an impact on this endpoint with manipulations employed in this work. Published reports indicate no change in global cholesterol level in male CYP46A1 knockout mice or in wildtype mice treated with efavirenz (Petrov et al., 2020). On the other hand, some viral manipulations of CYP46A1 result in cholesterol alterations (Ayciriex et al., 2017).

While in both species striatum had the highest CYP46A1 expression and cerebellum the lowest, the magnitude of CYP46A1 expression in cortex and hippocampus was reversed between human and mouse (Figures 1A, 2B). This may be, at least in part, explained by relatively close level of expression between cortex and hippocampus, the fact that different techniques were utilized to quantify CYP46A1 expression between the two species or potentially a genuine species difference. Nonetheless, it appears that CYP46A1 expression is relatively well-conserved between rodents and humans in brain areas examined suggesting that rodent species may be suitable for preclinical studies testing the impact of CYP46A1 inhibitors in human disease conditions. We note that although rat tissue was used for testing of the physiological impact of inhibitors (Figures 5B–D), rat vs. mouse CYP46A1 protein sequence is 98.6% identical and 99.6% similar (EMBOSS Needle alignment) at the amino acid level, and 24S-HC levels previously measured in rat are similar to those measured in mice in the present work (Quan et al., 2003).

Brain regions with higher 24S-HC, such as the striatum, cortex or hippocampus (Figure 2C), may be more affected by treatment with CYP46A1 inhibitors. To test this hypothesis, we applied two structurally distinct, potent, efficacious and selective CYP46A1 inhibitors (Figure 3) to mouse striatal and hippocampal slices for up to 6 h. We expected compounds 1 and 2 to reduce 24S-HC concentration, but this was not observed (Figure 4). Although negative results can be difficult to interpret, we do not believe that degradation of inhibitors is likely, as *in vivo* experiments showed good compound exposure for several hours (Figure 6). The result of Figure 4 could suggest a lack of an effect of the inhibitors for rodent CYP46A1 enzyme or tissue retention of previously synthesized 24S-HC.

We reasoned that we could address some of these potential explanations for lack of 24S-HC reduction with the CYP46A1 inhibitors through brain slice functional assessment. As such, we utilized hippocampal slice long term depression, a form of synaptic plasticity that we observed to be susceptible to CYP46A1 knockout and rescued through exogenous 24S-HC application (Figure 5A), to test for functional effects of compounds 1 and 2. Rat hippocampal slice treatment with voriconazole, a previously reported CYP46A1 inhibitor (Shafaati et al., 2010), and compounds 1 and 2 resulted in ablation of LTD, which was restored through exogenously applied 24S-HC, with two of the three compounds (Figure 5), consistent with inhibition of CYP46A1 and lowering of 24S-HC. Through this CYP46A1 sensitive functional readout we conclude that the inhibitors are likely impacting 24S-HC levels within half an hour post administration and are as functional on rodent CYP46A1 as they are on the human enzyme. Thus, 24S-HC tissue retention, in the context of the slice media, is likely responsible for lack of overall 24S-HC change. Our results also reinforce the previously raised hypothesis that CYP46A1, and by default 24S-HC, is compartmentalized intracellularly (Sodero et al., 2012). We speculate that newly synthesized 24S-HC is important locally for LTD induction, but globally retained 24S-HC is insufficient. Although local inhibition of NMDAR function may be the most likely mediator of LTD inhibition, we found no effect of any of the CYP46A1 inhibitors on isolated NMDAR EPSPs elicited at low frequency (Supplementary Figure 3). Thus, CYP46A1 mobilization may depend on sustained NMDAR stimulation required for plasticity induction (Sodero et al., 2012). Furthermore, despite the *in vitro* selectivity of compound 2 (Supplementary Tables 1–3), it is possible we encountered an off target mediated effect on LTD (Supplementary Table 1). A difference between compound 1 and compound 2 could arise from the substantial structural differences (Figure 1A). Cultivating inhibitors from differing structural scaffolds could be beneficial in the search for a clinically useful compound.

Several possibilities exist for an LTD, but not an LTP effect in hippocampal slices from CYP46A1 KO mice (Figure 5A; Sun et al., 2016a). It seems unlikely that oxysterol-dependent LXR effects are involved as changes to plasticity are rapidly induced by drugs, yet, transcriptional changes typically require time, and CYP46A1^–/–^ animals actually exhibit counterintuitive changes to LXR transcriptional targets (Mast et al., 2017). We have previously shown that CYP46A1^–/–^ hippocampal neurons exhibit reduced NMDAR tone (Sun et al., 2016a). Although 24S-HC NMDAR effects would be expected to modulate both LTP and LTD, LTD could exhibit higher sensitivity than LTP to small changes to NMDAR function triggered by changes in local 24S-HC concentration. Another possibility is that local NMDAR-induced changes to cholesterol at synapses, induced by activity-driven CYP46A1 catalysis (Sodero et al., 2012), could be important to LTD but not LTP. CYP46A1^–/–^ mice do not exhibit changes to brain bulk cholesterol concentration (Lund et al., 2003; Meljon et al., 2014), but local changes could be relevant. Finally, given that oxysterol signaling is still relatively poorly understood, the impact on LTD could involve an undescribed mechanism.

Systemic acute administration of compound 1 confirmed that 24S-HC reductions can be achieved (Figures 6B,C) with a CYP46A1 inhibitor, reinforcing our conclusions from the hippocampal slice experiments regarding *in vivo* utility of these compounds. However, we observed a lag in brain 24S-HC reduction relative to exposure of the inhibitor as the maximal compound exposure was observed 2 h after administration (Figure 6A) yet statistically significant brain 24S-HC reductions were noted only after 4, 8, and 16 h (Figure 6C). It is hypothesized that this delay, at least in part, may be a function of very high brain 24S-HC reservoir, brain clearance rate of 24S-HC and the rate at which new 24S-HC is synthesized. Furthermore, larger 24S-HC drawdowns were observed with a subchronic treatment of the two CYP46A1 inhibitors (Figures 6E,F,H,I) which enabled compound 1 exposure beyond CYP46A1 *in vitro* IC_50_ for about 8 h a day with the 30 mg/kg dose (Figure 6D). These results suggest that longer inhibition of CYP46A1, even when it is not constantly engaged, may lead to larger and more persistent overall 24S-HC reductions, as reported in the clinic (Bialer et al., 2018).

## Conclusion

In conclusion, our data support the hypothesis that inhibition of the CYP46A1 enzyme impacts brain physiology, most likely due to lowering 24S-HC levels locally to affect neuronal function. CYP46A1 inhibitors such as those characterized here could prove to be clinically relevant treatments in diseases associated with elevated 24S -HC or NMDAR dysfunction. Furthermore, our data suggest that rodents could be an adequate species to study the effects of CYP46A1 inhibition *ex vivo* and *in vivo*.

## Data Availability Statement

All datasets generated for this study are included in the article/Supplementary Material or can be accessed by contacting the authors.

## Ethics Statement

The studies involving human participants were reviewed and approved by the Genotype-Tissue Expression (GTEx) Project was supported by the Common Fund of the Office of the Director of the National Institutes of Health, and by NCI, NHGRI, NHLBI, NIDA, NIMH, and NINDS. The patients/participants provided their written informed consent to participate in this study. The animal study was reviewed and approved by the Institutional Animal Care and Use Committee of Shanghai ChemPartner Co., LTD., and the Washington University Institutional Animal Care and Use Committee.

## Author Contributions

MP, AH, CZ, and SMe designed the study. YI, JD, and H-JS ran experiments. MP and SMe wrote the manuscript. AH, JD, SMi, and CZ edited the manuscript. MP and SMe analyzed the results. AH synthesized compounds 1 and 2. All authors contributed to the article and approved the submitted version.

## Conflict of Interest

MP, AH, JD, and SMi were employees of Sage Therapeutics while this work was performed. Other authors were employed by Washington University in St. Louis School of Medicine. CZ was a member of the scientific advisory board for Sage Therapeutics and holds equity in Sage Therapeutics.
