# Supplementary material for: Effects of CYP46A1 Inhibition on Long-Term-Depression in Hippocampal Slices ex vivo and 24S-Hydroxycholesterol Levels in Mice in vivo
Source: Front Mol Neurosci. 2020 Oct 29;13:568641. doi: 10.3389/fnmol.2020.568641 (PMC7658267; doi:10.3389/fnmol.2020.568641)
Supplement: Supplementary file 1 [file Data_Sheet_1.PDF]

Supplementary Material

1 Supplementary Data

Supplementary Figure 1: 24S-HC brain expression is not impacted by rodent's sex. 24S-HC brain distribution was the same in males and females in the striatum (A), hippocampus (B), cerebellum (C) and cortex (D).

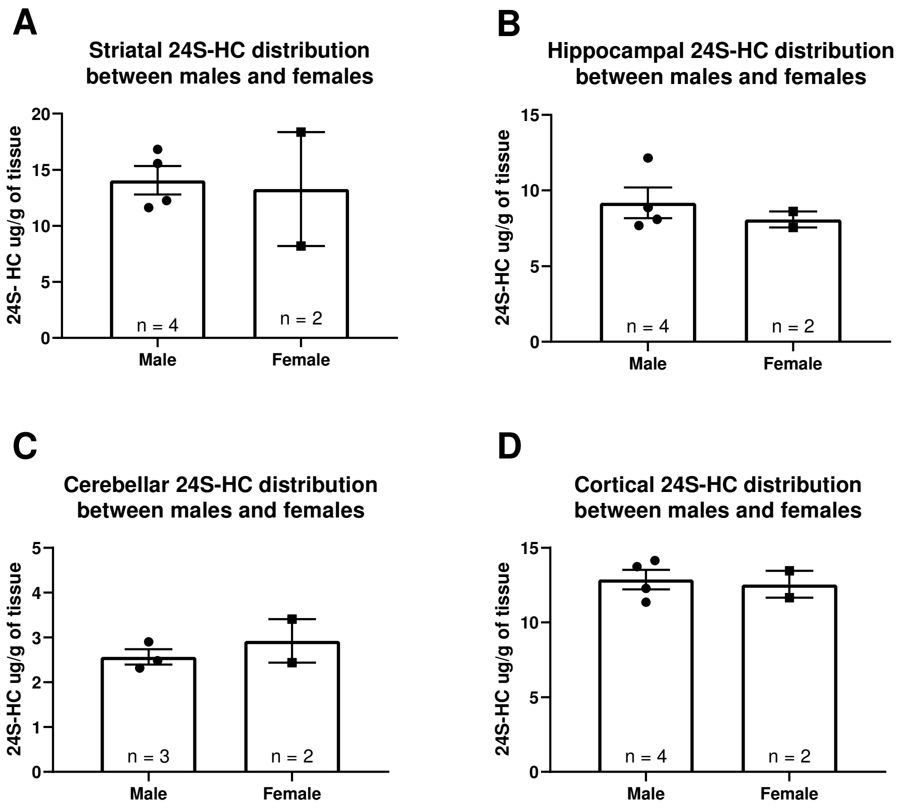

Supplementary Figure 2: Immunohistochemistry staining of hippocampal slices from WT and CYP46A1 KO mice with the #SAB-1410134; MilliporeSigma antibody, the antibody used for Western blots. The scale bar is 200 µm. Under isoflurane anesthesia, mice were perfused transcardially with 4% paraformaldehyde/0.2% glutaraldehyde in phosphate buffered saline. Brains were cryoprotected in 30% sucrose for 48 hours. Frozen 40 µm sections were cut on a freezing microtome. Free-floating sections were incubated in 1.5% normal goat serum, 1% bovine serum albumin, and 0.3% Triton X-100 for 1 h. Tissue was incubated in primary antibody at 1:1000 overnight with shaking at 4°C. Slices were incubated in goat anti-rabbit secondary antibody conjugated to Alexa Fluor 555 at a 1:300 dilution for two hours. Sections were mounted and photographed under epifluorescence. Several fixation, blocking, and antibody concentration variation protocols were tried with similar results

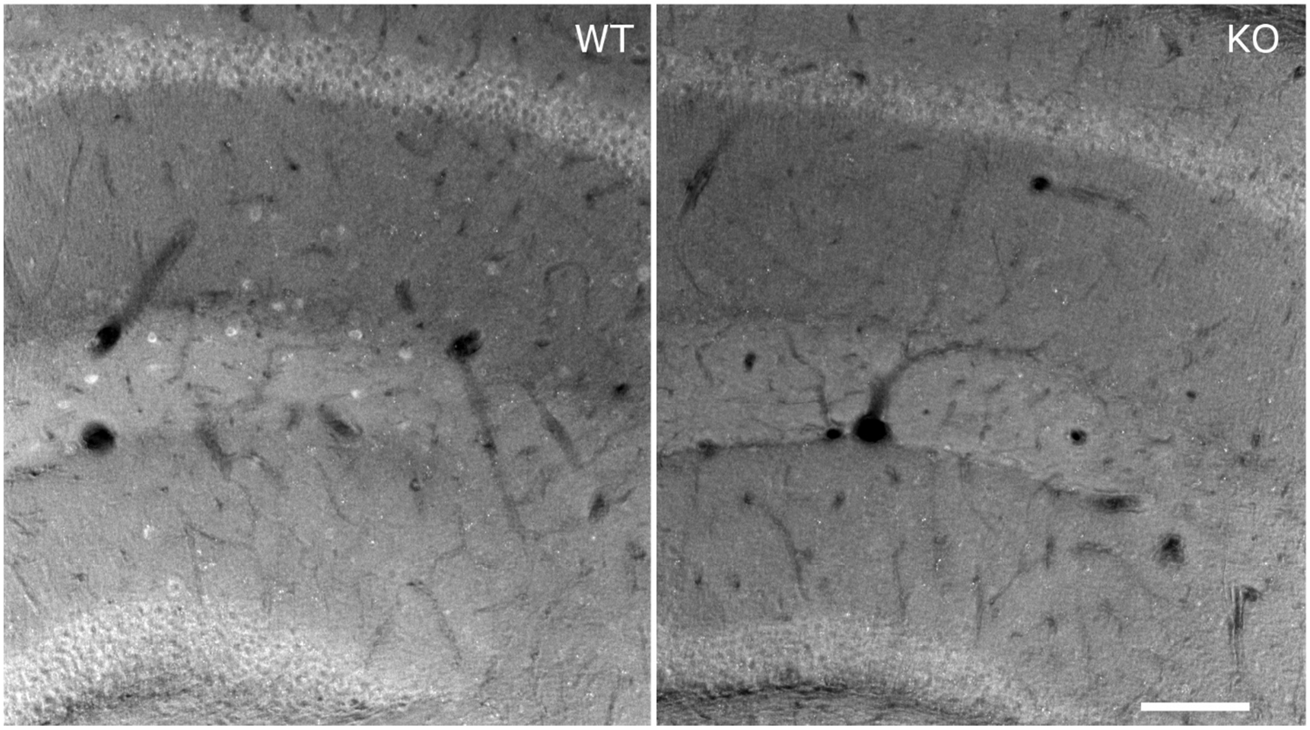

Supplementary Figure 3: NMDAR EPSPs are unaffected by CYP46A1 inhibitors. EPSPs were elicited every minute in low bath Mg^{2+} (0.1 mM) and CNQX (30 uM). Drugs were added for the times indicated by the horizontal bars at 10 µM (Compound 1) or 1 µM (Compound 2). Voriconazole was applied in escalating concentrations as indicated. Insets show sample traces.

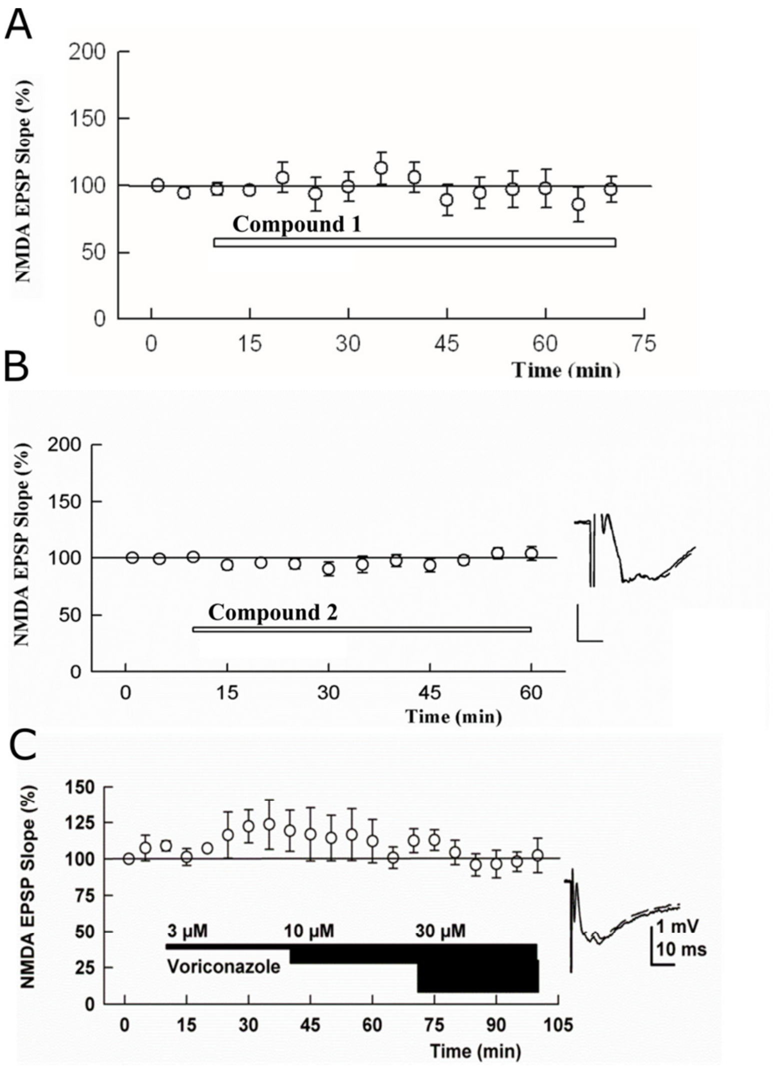

Supplementary Table 1: I*n vitro* off-target profile of CYP46A1 inhibitors. CEREP binding assay with the two CYP46A1 inhibitors tested at 10 μM (n = 2) did not meaningfully bind to any of the off-target protein panel.

	compound 1	Compound 2
Human A1	32%	12.2%
Human A2A	1.1%	-2.6%
Human A3	23.3%	5.4%
α1	7.8%	6.1%
α2	-0.5%	-10.7%
Human β1	-3.2%	-0.7%
Human β2	-8.1%	-2.0%
Human AT1	-3.7%	-3.2%
BZD	3.7%	-3.3%
Human B2	6.9%	8.4%
Human CB1	-34.7%	-9.2%
CCK1	7.1%	-4.7%
Human D1	2.4%	-5.3%
Human D2s	-15.2%	-15.4%
Human Eta	-25.8%	-35.5%
GABA	0.7%	6.5%
GAL2	-7.2%	0.0%

AMPA	-5.6%	-1.5%
Kainate	-2.4%	-19.9%
NMDA	4.7%	-5.3%
Glycine	-2.8%	-8.3%
Human CXCR2	-11.3%	-1.5%
Human CCR1	2.6%	-1.7%
Human H1	-12.9%	-13.3%
Human H2	-6.2%	-5.4%
Human H3	-3.3%	-14.7%
Human MC4	4.5%	9.8%
MT1	1.7%	8.3%
Human M1	3.3%	-8.6%
Human M2	03.7%	12.3%
Human M3	-9.1%	7.3%
Human NK2	-7.9%	-1.4%
Human NK3	4.1%	1.1%
Human Y1	-2.9%	-16.3%
Human Y2	-3.7%	6.8%
Human NTS1	-9.7%	-12.2%
Human DOP	-6.2%	-5.8%

Human KOP	8.0%	-5.2%
Human MOP	-0.7%	2.5%
Human NOP	1.4%	0.6%
PCP	-3.0%	4.9%
Human EP4	-22.4%	1.8%
P2X	5.8%	6.1%
P2Y	-13.4%	5.7%
Human 5-HT1A	12.4%	10.8%
Human 5-HT1B	-8.4%	-13.3%
Human 5-HT2A	-3.2%	-5.0%
Human 5-HT2B	-2.8%	-2.4%
Human 5-HT3	11.0%	0.8%
Human 5-HT5A	-12.5%	9.6%
Human 5-HT6	-0.7%	0.9%
Human 5-HT7	5.4%	4.2%
Human sigma	19.2%	-9.0%
SST	-15.7%	-19.1%
Human GR	-8.6%	-0.6%
Human PR	2.0%	-4.7%
Human PXR	4.8%	8.8%

Human AR	-2.0%	3.1%
Human VPAC1	5.5%	-1.9%
Human V1a	-7.4%	2.8%
Ca2+ channel	3.7%	9.4%
Kv channel	-5.8%	-6.8%
SKca channel	-12.0%	-13.1%
Na+ channel	-10.7%	-6.2%
Cl- channel	16.3%	6.4%
Human norepinephrine transporter	-14.5%	-3.6%
Human dopamine transporter	2.1%	13.8%
GABA transporter	1.6%	-11.1%
Human 5-HT transporter	1.2%	0.1%

Supplementary Table 2: No CYP off-target pharmacology with the two tool compounds.

	compound 1	compound 2
CYP1A2	6.29 µM	> 50 µM
CYP2B6	> 50 µM	> 50 µM
CYP2C19	> 50 µM	> 50 µM
CYP2C8	> 50 µM	> 50 µM
CYP2C9	> 50 µM	> 50 µM

CYP2D6	> 50 μM	> 50 μM
CYP3A4_M	> 50 μM	> 50 μM
CYP3A4_T	> 50 μM	> 50 μM

Supplementary Table 3: No off-target kinase activity with the two tool compounds tested at 1 μM in a 90-kinase panel.

Target	compound 1	compound 2
Gene Symbol	%Ctrl @ 1000nM	%Ctrl @ 1000nM
ABL1(E255K)-phosphorylated	84	66
ABL1(T315I)-phosphorylated	88	86
ABL1-nonphosphorylated	70	72
ABL1-phosphorylated	71	58
ACVR1B	90	96
ADCK3	100	100
AKT1	99	100
AKT2	99	100
ALK	67	66
AURKA	100	84
AURKB	100	95
AXL	71	87
BMPR2	100	81
BRAF	87	80
BRAF(V600E)	96	94
BTK	97	91
CDK11	94	99
CDK2	81	91
CDK3	94	92
CDK7	92	87
CDK9	99	89
CHEK1	95	92
CSF1R	100	100
CSNK1D	86	87
CSNK1G2	95	94
DCAMKL1	83	71
DYRK1B	100	100
EGFR	100	100
EGFR(L858R)	100	100
EPHA2	92	89
ERBB2	72	72
ERBB4	100	91
ERK1	99	98
FAK	98	98
FGFR2	99	100
FGFR3	100	97
FLT3	72	86
GSK3B	99	74
IGF1R	99	98
IKK-alpha	99	91
IKK-beta	90	76
INSR	95	98
JAK2(JH1domain-catalytic)	80	87
JAK3(JH1domain-catalytic)	90	94
JNK1	81	76
JNK2	87	65
JNK3	87	78
KIT	100	88

KIT(D816V)	92	91
KIT(V559D,T670I)	99	97
LKB1	96	100
MAP3K4	100	100
MAPKAPK2	76	89
MARK3	74	75
MEK1	91	84
MEK2	91	90
MET	100	100
MKNK1	81	76
MKNK2	77	61
MLK1	93	98
p38-alpha	100	96
p38-beta	86	98
PAK1	100	100
PAK2	100	100
PAK4	100	99
PCTK1	90	79
PDGFRA	98	99
PDGFRB	100	85
PDPK1	97	99
PIK3C2B	100	100
PIK3CA	100	100
PIK3CG	100	97
PIM1	100	95
PIM2	97	100
PIM3	100	94
PKAC-alpha	95	96
PLK1	73	73
PLK3	85	68
PLK4	100	81
PRKCE	63	61
RAF1	97	94
RET	87	99
RIOK2	100	93
ROCK2	100	96
RSK2(Kin.Dom.1-N-terminal)	100	98
SNARK	86	80
SRC	90	93
SRPK3	95	96
TGFBR1	98	99
TIE2	100	100
TRKA	99	99
TSSK1B	100	100
TYK2(JH1domain-catalytic)	99	100
ULK2	94	69
VEGFR2	94	96
YANK3	81	100
ZAP70	100	100
